# Inferring the perturbed microRNA regulatory networks from gene expression data using a network propagation based method

**DOI:** 10.1186/1471-2105-15-255

**Published:** 2014-07-29

**Authors:** Ting Wang, Jin Gu, Yanda Li

**Affiliations:** MOE Key Laboratory of Bioinformatics, TNLIST Bioinformatics Division & Center for Synthetic and Systems Biology, Department of Automation, Tsinghua University, Beijing, 100084 China

**Keywords:** MicroRNA, Gene regulatory networks, Network analysis, Gene expression, Cancer

## Abstract

**Background:**

MicroRNAs (miRNAs) are a class of endogenous small regulatory RNAs. Identifications of the dys-regulated or perturbed miRNAs and their key target genes are important for understanding the regulatory networks associated with the studied cellular processes. Several computational methods have been developed to infer the perturbed miRNA regulatory networks by integrating genome-wide gene expression data and sequence-based miRNA-target predictions. However, most of them only use the expression information of the miRNA direct targets, rarely considering the secondary effects of miRNA perturbation on the global gene regulatory networks.

**Results:**

We proposed a network propagation based method to infer the perturbed miRNAs and their key target genes by integrating gene expressions and global gene regulatory network information. The method used random walk with restart in gene regulatory networks to model the network effects of the miRNA perturbation. Then, it evaluated the significance of the correlation between the network effects of the miRNA perturbation and the gene differential expression levels with a forward searching strategy. Results show that our method outperformed several compared methods in rediscovering the experimentally perturbed miRNAs in cancer cell lines. Then, we applied it on a gene expression dataset of colorectal cancer clinical patient samples and inferred the perturbed miRNA regulatory networks of colorectal cancer, including several known oncogenic or tumor-suppressive miRNAs, such as miR-17, miR-26 and miR-145.

**Conclusions:**

Our network propagation based method takes advantage of the network effect of the miRNA perturbation on its target genes. It is a useful approach to infer the perturbed miRNAs and their key target genes associated with the studied biological processes using gene expression data.

**Electronic supplementary material:**

The online version of this article (doi:10.1186/1471-2105-15-255) contains supplementary material, which is available to authorized users.

## Background

MicroRNAs (miRNAs), a class of ~22 nt endogenous small regulatory RNAs, can induce the degradation or translational repression of mRNA transcripts through sequence-specific binding to their 3’-UTRs [1, 2]. To date, many miRNAs and their target genes have been found to play important roles in various biological processes. The dys-regulations or perturbations of miRNA regulatory networks are closely related to many cellular phenotype changes and diseases [3, 4]. Identifications of the perturbed miRNAs regulatory networks are important for understanding the molecular mechanisms of the studied biological processes.

To study miRNA functions, biologists usually overexpress or knockdown specific miRNAs in cells and observe their impacts on cellular states and functions [5, 6]. The miRNA regulatory networks are usually cell-type specific [4], which makes it impractical to test and verify all miRNAs in all cellular conditions due to the high experimental cost. Currently, most miRNA-target annotations come from sequence-based predictions without cell-type or condition specific information [7]. Therefore, some computational methods are developed to infer the perturbed miRNAs regulatory networks associated with specific phenotype changes by integrating the sequence-based miRNA-target predictions [8–10] with the high throughput genome-wide gene expression data. One popular method is gene set enrichment analysis (GSEA), which determines whether a pre-defined set of genes show statistically significant, concordant differences between two biological states or phenotypes [11]. The hypothesis is that if the expressions of the miRNA targets are significantly changed, the corresponding miRNA should be aberrant or perturbed in the studied process [12]. In addition, miRNAs generally fine-tune the expression of target genes [13–15]. The methods (such as GSEA) which only consider the expression changes of the direct target genes frequently fail to identify the perturbed miRNA regulatory networks. The intracellular system can be regarded as a complex molecular network, some studies combine the network information and the expression data to improve prediction performances [16]. For example, GeneRank algorithm takes gene expression importance into account and employs random walk on gene-gene interaction network to re-score all genes [17]. The new score better reflects the systematic importance of genes in cells and it can also be used to analyze miRNA target set enrichments. However, the gene expression changes should be the responses of driver perturbations on the global gene regulatory networks: when a miRNA is perturbed, it will firstly impact its direct targets and subsequently affect the expression of the downstream genes through intracellular molecular regulatory networks, and finally change the global gene expression patterns in cells. Therefore, a network propagation based model should be more reasonable for interpreting the global transcriptional response to miRNA perturbations than the methods only considering the differential information of miRNA target genes.

In this study, we proposed a network propagation based method (NP-method) to identify the perturbed miRNA regulatory networks from the gene expression data. It used random walk with restart [18, 19] in gene regulatory networks to estimate the global network effect of miRNA perturbation on its direct target genes, and meanwhile use a forward searching strategy [20] to find the key target genes regulated by the perturbed miRNAs, which are most likely to generate the observed global gene expression changes. We tested it on several gene expression datasets generated from miRNA overexpression or knockdown experiments. Resuls show that it can better rediscover the perturbed miRNAs than several compared methods. Then it was used to infer the perturbed miRNA regulatory networks in colorectal cancer from a gene expression dataset of clinical patient samples. Several known oncogenic and tumor-suppressive miRNAs, including miR-17, miR-26 and miR-145 were identified by NP-method.

## Methods

### Overview

**Figure 1:**
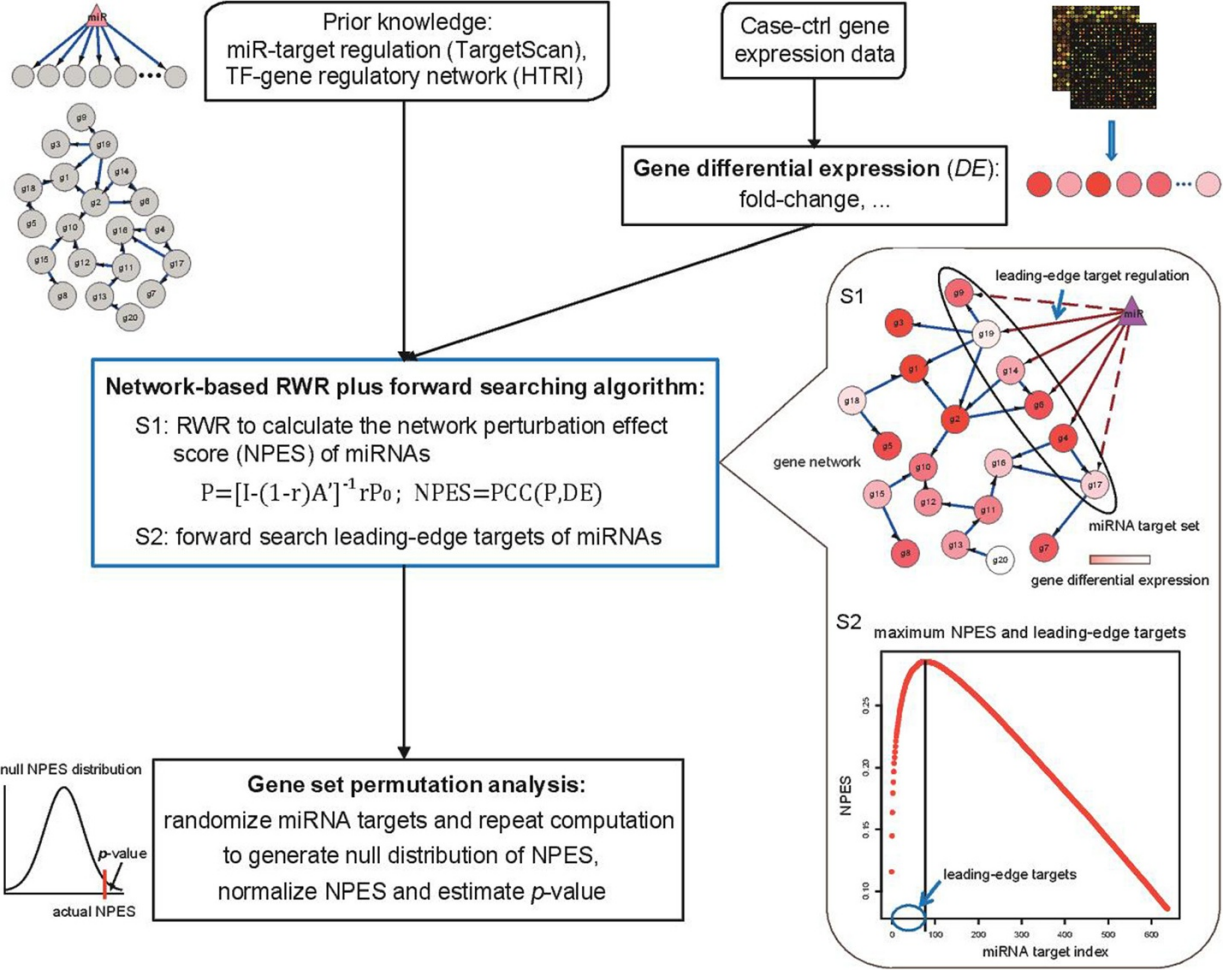
**Overview of the network propagation based method to infer the perturbed miRNA regulatory networks from gene expression data.**

The network propagation based method (NP-method) is developed to infer the key miRNA regulatory networks whose perturbation is most likely to induce the observed global gene expression changes (See workflow in Figure 1). By integrating gene differential expression information with biological prior knowledge, such as the miRNA-target regulations and the TF-gene regulatory network, a novel network-based random walk with restart (RWR) plus forward searching algorithm is carried out to calculate the network perturbation effect score (NPES) of miRNAs and extract their leading-edge target genes. Gene set permutation analysis is implemented to normalize the score and estimate the *p*-value for each miRNA. The software is freely available at [21].

### Materials

#### Gene expression profiles

To verify the efficiency of NP-method in identifying perturbed miRNAs, we analyzed seven case-ctrl gene expression datasets, which were generated from the miRNA overexpression or knockdown experiments, and one of them was a time-course data involving seven time-point gene expression observations (Table 1). We also applied the method on a cancer-normal gene expression dataset to infer the perturbed miRNA regulatory networks in colorectal cancer. All raw microarray data or series matrixes were downloaded from the Gene Expression Omnibus (GEO) [22]. These raw data were firstly quantile-normalized with the robust multichip average (RMA) method [23]. All gene expression values were transformed into log2 scale and their IDs were mapped into Entrez Gene IDs [24].

**Table 1:** **Gene expression data analyzed in this work**

Dataset	Cell	miRNA	Treatment	Sample
GSE33420	CRC (DLD-1)	miR-143	Overexpression	4 case + 4ctrl
GSE18625	CRC (DLD-1)	miR-145	Overexpression	4 case + 3ctrl
GSE7754	CRC (HCT116)	miR-34a	Overexpression	2 case + 2ctrl
GSE16568	OVCA (ES-2)	miR-22	Overexpression	3 case + 3ctrl
GSE16569	OVCA (OVSAYO)	miR-30a/30d	Knockdown	3 case + 3ctrl
GSE16572	OVCA (ES-2)	miR-182	Knockdown	3 case + 3ctrl
GSE6207	HCC (HepG2)	miR-124	Overexpression	Case-ctrl, time-course (4 h, 8 h, 16 h, 24 h, 32 h, 72 h, 120 h)
GSE4107	Colonic mucosa	NA	NA	12 cancer + 10 normal

#### Prior molecular regulation information

It is well known that some miRNAs belong to the same families with the same seed sequence, which is typically defined as position 2–8 from the 5' end of a mature miRNA and is very important for deciding which targets the miRNA regulates [25]. The miRNAs within the same families may regulate similar targets and are often thought to have interrelated or redundant functions [25, 26]. So we focused our study objects on the miRNA families, which could also reduce the number of candidates and thus be better for the multiple testing correction in statistics [27]. Therefore, for the miRNA-target regulation information, We collected the conserved targets of 153 miRNA families from the widely-used miR-target prediction database TargetScan v6.2 [8].

For the gene regulatory network information, we employed and compared two networks. One is a high-quality human gene transcriptional regulatory network, which comes from an open-access database of experimentally verified human transcriptional regulation interactions – HTRIdb [28]. This network contains 18,310 nodes and 51,871 directed edges. The other one is a protein-protein interaction (PPI) network, which comes from the PPIs scored higher than 0.9 in database STRING v9.0 [29]. This network contains 9,598 nodes and 57,326 edges, and is often used as a highly-reliable PPI network in systems or network biology. However, it is known that prior network knowledge usually contains some noises. To discuss the influence of the noisy edges, we randomly added and deleted 10% edges in the TF-gene regulatory network.

### Methods

#### Random walk with restart from miRNA targets for modeling the network effect of miRNA perturbations

In viewpoint of network biology, perturbation of a miRNA firstly impacts its direct targets, and then the effect will propagate through intracellular molecular networks and ultimately influence the expression of all genes in cells (Figure 1 and Additional file 1: Figure S1). The exact gene regulatory parameters are unavailable, so we utilized a method named random walk with restart (RWR) to make use of the network topology for estimating the network effect of miRNA perturbations [18].

Assume that a gene regulatory network *G* contains *N* genes, and an adjacent matrix *A* with N*N dimension represents the gene regulatory interactions. *A*_*ij*_ = 0 means no interaction between gene *i* and gene *j*. For the transcriptional regulation network, *A* is an unsymmetrical matrix where *A*_*ij*_ = 1 means gene *j* regulates gene *i*. To make it nonsingular and reversible, we set its diagonal elements as 1*e*-10. While for the PPI network, A is symmetrical and *A*_*ij*_ = *A*_*ji*_ = 1 means gene *i* and gene *j* interact with each other. Each column of *A* was firstly scaled to have sum 1, and this produced a normalized adjacent matrix *A’*.

Besides, suppose a miRNA that has *x* targets is perturbed, then the influence will spread across the network starting from the target genes. In our RWR model, a random walker starts from the *x* seed nodes (i.e. the miRNA targets) in network *G* with an initial probability distribution *P*_*0*_, whose length is *N* and elements corresponding to the seed nodes are equally set as 1/*x* while the others are 0. The walker appears in the network with a probability distribution *P* following an iterative rule as Eq. (1): at each step, the walk is decided iteratively by a Markov chain with a probability transition matrix *A’*, and the restart of the walk at the seed nodes is allowed with a restart probability *r*.
1

When the system becomes stable and the *P* is convergent, which means *P*^*n*+1^=*P*^*n*^, so the steady-state probability distribution *P* can be directly worked out as below without the time-consuming iteration steps:
2

Here *P* represents the probability of each gene in the network to be perturbed when the cell gets stable. The expression of the gene with larger *p* is more likely to be influenced by the miRNA perturbation. Under this hypothesis, we calculated a network perturbation effect score (NPES) for the miRNA, which is defined as the Pearson correlation coefficient (PCC) between the global gene perturbed probabilities (*P*) and the corresponding gene differential expression levels (*DE*):
3

Here *DE* can be any measure of the gene differential expressions between two biological situations, such as fold-change, *t*-statistic or *z*-score, and it is transformed into the absolute value. *N* is the size of *P* and *DE*.  and  are the mean values. The score *NPES* quantifies the degree of miRNA-induced gene perturbed probabilities matching gene differential expression levels. The larger the score is, the better the miRNA interprets the observed gene expression changes.

#### Forward search the leading-edge targets of miRNAs

Averagely, a miRNA have hundreds of predicted targets, but not all of them are regulated in a specific cellular condition, and the same miRNA may regulate different subsets of targets under different conditions. Therefore, uncovering the key miRNA targets with relation to specific conditions is very important for understanding the function and regulatory mechanism of a miRNA. In this study, we borrowed the concept of leading edge subset of genes introduced by GSEA, which is a small group of genes in a specified gene set that can generate a maximal enrichment score to evaluate the differential expression of the gene set [11], and defined these key targets of a miRNA to be its leading-edge (LE) targets, which can maximize the *NPES* score and best explain the observed gene expression changes for the specified miRNA.

In our method, miRNA targets are regarded as the RWR seeds, so identifying the LE targets is actually optimizing the seed set to generate a best network perturbed probability *P* that can maximize the *NPES*. Here we propose a forward searching strategy to achieve this goal. Note in Eq. (2) *M* depends on the network adjacent matrix *A* and the RWR restart probability *r*. When they are fixed, *M* will be a constant matrix and the steady-state probability *P* will only depend on the initial probability *P*_*0*_, which is decided by the seeds. Thus to search the LE targets turns out to optimize the *P*_*0*_. Our searching procedures are shown as follows (given a miRNA with *x* targets):

1. Let each target be the RWR seed at each time and calculate the corresponding *NPES*, then get a score vector *[NPES*_*1*_*, NPES*_*2*_*, …, NPES*_*x*_*]*;
2. Sort this score vector in descending order and sort targets accordingly, then get a target rank *[t*_*(1)*_*, t*_*(2)*_*, …, t*_*(x)*_*]*;
3. Start from the first target in the rank and add the rest one by one to compose new RWR seed sets and calculate the corresponding *NPES*s, then get a new score vector *[NPES*_*1*_*', NPES*_*2*_*', …, NPES*_*x*_*']*;
4. Extract the maximum score and the corresponding seed set to get the final *NPES* and the LE targets of the miRNA (Figure 1 S2).

#### Gene set permutation analysis to normalize NPES and estimate p-value

To avoid producing bias towards the miRNAs with large target set, we performed a permutation-based statistical analysis to normalize the *NPES* and assess its statistical significance. The gene labels of miRNA targets were randomly assigned from whole network genes, and then a group of new scores were calculated using the randomized miRNA target sets through all the above steps. This process was repeated several times (e.g. 1,000) to generate null distribution of the *NPES* for each miRNA.

Subsequently, we computed the empirical *p*-value for the score of each miRNA, which is the proportion of obtaining *NPES* in the null distribution not less than the one actually observed [30]. We implemented the false discovery rate (FDR) multiple testing correction to adjust the *p*-values of all miRNAs with the Benjamini & Hochberg method [27] using a widely used R package “p.adjust”. In addition, to eliminate the set size effect, we normalized *NPES* as a *z*-score:
4

Here the mean and standard deviation were calculated from the null distribution. Then the scores of different miRNAs were comparable, larger score implied the miRNA took more responsibility for the observed gene expression changes and should be more important for the studied biological process. We finally ranked miRNAs according to the normalized scores.

#### Comparisons with other methods

We compared NP-method with two other methods on predicting the perturbed miRNAs. One is the popular gene set enrichment analysis (GSEA), which determines whether an a priori defined set of genes shows statistically significant, concordant differences between two biological states or phenotypes [11]. We used software GSEA v2.0.14 Java version to analyze the differential expression of each miRNA’s target set and estimate the activity of corresponding miRNA. GSEA only uses the gene expression information, while the other method, termed GR.GSEA, further integrates gene-gene network information. It firstly applies the GeneRank algorithm to re-score all genes by using both gene differential expression and gene network information [17], then uses the new gene scores to execute GSEA and estimate the miRNA activities.

During the analysis of gene expression data coming from miRNA overexpression or knockdown experiments, we sorted miRNAs in descending order according to the normalized scores (i.e. the *NPES*_*zscore*_ in NP-method, the normalized enrichment score in GSEA and GR.GSEA generated by the GSEA software), and compared these methods using the putative rank of the experimentally perturbed miRNAs. If the desired miRNA is ranked at the top, it implies the corresponding method can predict well enough. While analyzing the gene expression data from CRC patient, we used the area under the receiver operating characteristic (ROC) curve, named AUC, to evaluate the prediction of cancer associated miRNAs. Larger AUC means better prediction [31]. For this analysis, we extracted those miRNAs associated with CRC from a miRNA-disease relationship database called miR2Disease to be gold standard miRNAs [32].

## Results

### Rediscovering the experimentally perturbed miRNAs from gene expression data

To verify the efficiency of identifying the perturbed miRNAs, we firstly applied our method on several gene expression datasets generated from miRNA overexpression or knockdown experiments (Table 1), and tried to rediscover the experimentally perturbed miRNAs through data analysis. We firstly calculated gene expression fold changes to be the gene differential expression inputs, and then estimated the network perturbation effect for each miRNA. We compared the putative rank of each experimented miRNA using different *r* in NP-method. When *r* = 0, *P* will be independent on *P*_*0*_, which means the perturbation effect will be determined only by the network topology and there will be no difference between any miRNAs; while when *r* = 1, *P* will always be *P*_*0*_, which means a miRNA only influences its target genes and there will be no network effect. So we tested *r* = 0.1, 0.2, …, 0.9. The results show little differences (Additional file 1: Table S1), so we used *r* = 0.5 as default in this study, which intuitively means that a miRNA’s impact is half on its direct targets and half on other genes through the network propagation. We compared the results of inferring the perturbed miRNAs by using the three different methods, and found that NP-method nearly always ranked the desired miRNAs better than the other two methods (See the first three columns in Table 2, more details can be found in Additional file 2). GSEA studies the expression of miRNA target set without considering the influence of gene-gene interactions, so it is not comprehensive enough to interpret the cellular gene expression responses after miRNA perturbations. For the GR.GSEA, although GeneRank integrates network information to reprioritize genes, it performs not so well as NP-method. The latter is more consistent with the nature of intracellular molecular regulatory mechanism and is a better model for explaining the miRNA-induced global gene expression changes. In addition, the results of NP-method using HTRI, HTRI10%add and HTRI10%del networks shows little differences, which imply that our method is robust in the transcriptional regulation network even with a few noisy edges. While using the STRING network, the method ranked these perturbed miRNAs not as good as that using the HTRI network (See the last four columns in Table 2). Therefore, we recommend the HTRI network, which should be more appropriate for analyzing intracellular gene expression regulations than the PPI network, in the future applications of NP-method.

**Table 2:** **Results of inferring the experimentally perturbed miRNAs using different methods and different networks**

Datasets	GSEA	GR.GSEA	NP-method			
			HTRI	HTRI10%add	HTRI10%del	STRING
GSE33420.CRC.mir-143	^a^1 (*)	1 (*)	1 (*)	1 (*)	1 (*)	1 (*)
GSE18625.CRC.mir-145	26 (0.033)	19 (0.035)	1 (*)	2 (*)	1 (*)	1 (*)
GSE7754.CRC.mir-34a	98 (0.672)	80 (0.796)	45 (0.484)	40 (0.539)	47 (0.503)	122 (0.99)
GSE16568.OVCA.mir-22	5 (*)	5 (*)	1 (*)	1 (*)	1 (*)	1 (*)
GSE16569.OVCA.mir-30a/30d	77 (0.004)	66 (0.245)	18 (0.011)	30 (0.037)	15 (0.01)	46 (0.192)
GSE16572.OVCA.mir-182	15 (*)	24 (0.044)	5 (0.008)	3 (0.005)	4 (0.007)	9 (0.03)

^a^Putative rank of the perturbed miRNA according to the normalized score (*p*-value of the miRNA); *< 0.001.

Except for detecting the perturbed miRNAs, NP-method can also identifies their key targets, called leading-edge (LE) targets, which are most likely regulated by the perturbed miRNAs in the specified condition and give rise to the observed gene expression changes. Take the CRC dataset GSE18625 for example, it found 49 LE targets for miR-145 in the transfected DLD-1 cells. Among them the *Src* family member *YES1* has been reported as a direct miR-145 target that plays oncogenic function in colon cancer [33], *FSCN1* and *PPP3CA* are also directly regulated by this miRNA in esophageal squamous cell carcinoma and urothelial carcinoma [34, 35]. Hence miR-145 may induce the observed transcriptional responses primarily through this regulatory sub-network. Since GSEA can also extract LE subset of genes, we used Fisher’s exact test to analyze their enrichments of validated miR-145 targets that are also related to colorectal cancer. To obtain a gold standard gene list for this analysis, we firstly collected 89 validated miR-145 targets from miRTarBase [36], which is one of the most comprehensive databases of experimentally validated miRNA-target interactions in various cells. Then we employed a literature mining approach to capture the genes associated with CRC: we automatically downloaded all PubMed abstracts related to a query of “(colon OR colorectal) AND cancer” using the NCBI Entrez E-Utilities and captured 5,943 unique genes/proteins. By intersecting these two gene sets, we obtain 58 gold standard genes that are proved direct targets of miR-145 and also functionally related to CRC. In the end, the LE target set extracted by our NP-method is significantly enriched with the gold standard genes although its size is small, while the *p*-values of other methods’ LE target sets are not significant (Table 3). These indicate that our method can efficiently identify the authentic and functional targets of the perturbed miRNAs. In fact, the NP-method always outputs less LE targets than the GSEA-based methods (Additional file 1: Figure S1), but it is more convenient for the scientists to select candidate miRNA targets for experimental dissection.

**Table 3:** **Enrichment results of validated and also CRC related miR-145 targets in the LE target sets**

	NP-method.LE	GSEA.LE	GR.GSEA.LE	miR-145.target
Validated	6	12	12	25
Other	43	216	282	655
Total	49	228	297	680
	^a^ *p*-value = 0.014	^a^ *p*-value = 0.194	^a^ *p*-value = 0.204	

^a^ *P*-value result of Fisher’s exact test using a contingency table integrating the data of the current and the 4th columns, it indicates the statistical significance of the current LE target set enriching with larger proportion of validated and functional miR-145 targets than the background target set.

**Figure 2:**
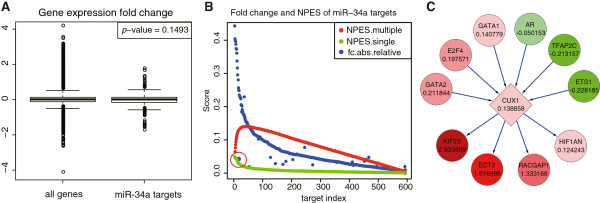
**A case study on the miR-34a overexpressed HCT116 cells. (A)** Fold change of all genes and miR-34a targets. The *p*-value is estimated by K-S test. **(B)** Fold change and *NPES* score of miR-34a targets. Blue dot represents the absolute value of gene expression fold change, which is normalized by the maximum of all genes; Green dot represents the *NPES* computed by using single target as the RWR seed in the step 1 of forward searching, and all the miRNA targets are sorted according to this score; Red dot stands for the *NPES* generated by using multiple targets as the RWR seed, the peak value is the optimal score and those targets appearing at and before this point are the leading-edge targets. **(C)** Neighbor sub-network of the miR-34a target *CUX1*. Gene name and expression fold change are labeled in each node.

It is known that miRNAs tend to fine-tune the expressions of genes [13, 14], and some miRNAs may regulate some targets only at protein level but not mRNA level [37, 38]. Considering the systematic propagation effect, the impact of miRNA perturbation on target genes could be explained by neighbor genes, so the NP-method should be appropriate for the condition that the expressions of miRNA targets are not markedly changed but the downstream genes are. Take the dataset GSE7754 as an example, by comparing gene expressions of the HCT116 cells with and without enforced miR-34a expression we found that the fold change of miR-34a targets were too indistinctive to be distinguished from the background genes (Figure 2A). From the putative ranks of miR-34a in Table 2, we see that the GSEA-based methods hardly predict this miRNA but NP-method performs much better. Figure 2B shows the fold changes and *NPES*s of the miR-34a targets. According to the multi-target-based *NPES*s (red dots), we extracted 36 leading-edge targets that appear at and before the peak point. In the figure we see a special LE target (*CUX1*), whose fold change is small (marked in a red circle) but *NPES* is relatively large. To illustrate the network perturbation effect of this target gene, we investigated its surrounding gene regulatory network (Figure 2C), where only *CUX1* was target of miR-34a. And we found that *CUX1* had a small fold change (0.138858, in log2 scale) but three downstream genes (*KIF23*, *ECT2* and *RACGAP1*) had remarkable fold changes. Besides, *CUX1* is a homeodomain transcriptional regulator known to be involved in the development and cell cycle progression, and its activity is associated with increased migration and invasiveness in numerous tumor cell lines including HCT116 or resistance to apoptosis in pancreatic cancer [39, 40]. And some other studies have reported that *KIF23*, *ECT2* and *RACGAP1* play important roles in the cell cycle and cell proliferation [41, 42]. These findings indicate that miR-34a can regulate the cancer process in an indirect and inconspicuous way, and it can be discovered only by our NP-method.

### Analyzing time-course gene expressions in HepG2 cells transfected with miR-124

Since NP-method can identify key target genes of miRNAs, exploring the similarities and differences among the key targets of the same miRNA under different situations can further help to understand roles of miRNAs in different context. Besides, it is said that the influence of miRNA perturbation on gene expression is time-dependent [43]. To check this and further test our method, we applied it on a time-course gene expression dataset from a miRNA transfection experiment (GSE6207). In detail, pre-miR-124 and negative control miRNA duplex were transfected into HepG2 cell line using the Reverse Transfection protocol recommended by Ambion, then the paired gene expressions at 7 time points (4, 8, 16, 24, 32, 72, 120 h) were measured using Affymetrix HG-U133Plus2 microarray platform [44]. To avoid noise signals, we firstly filtered the low-expressed genes using a rank-based strategy: the genes whose expression values ranked at the lowest 20% in more than 80% samples were removed. This process generated an expression profile containing 15,444 genes, whose fold changes at each time point were then calculated to be the differential expression inputs of the three methods.

**Figure 3:**
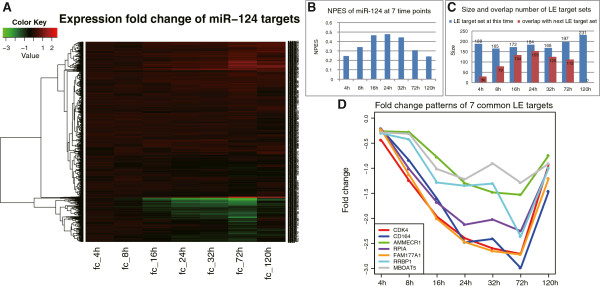
**Analysis on the time-course data of miR-124 transfection. (A)** Clustering diagram of miR-124 target expression fold changes. **(B)** *NPES* of miR-124 at each time point. **(C)** Size and overlap number of the LE target sets of miR-124. Blue bar represents the LE target set at current time point; red bar represents the overlap between the LE target sets of current and next time point. **(D)** Fold change patterns of the 7 common LE targets of miR-124 that appear at every time point.

Results demonstrate that NP-method ranks miR-124 much better than GSEA and GR.GSEA at 4 h and slightly better than them at 120 h, and in the middle period all methods perform very good (Table 4). According to the prediction of miRNA-target interactions in TargetScan [8], there are 1,564 conserved target genes for miR-124 family. Figure 3A shows the clustering diagram of the expression fold change of all miR-124 targets, from which we see at 4 h after miRNA transfection the expressions of targets vary not much, and as time goes on more and more targets’ expressions are markedly repressed, while at 120 h their differential expressions return indistinctive. Maybe at the very beginning (4 h) the transfected miRNA cannot rapidly and greatly affect the mRNA concentrations of target genes, but their protein translations are directly repressed and further influence other genes within network, so the NP-method performs much better than the other two methods due to its innovative consideration on the network propagation effect. However, after five days (120 h) the influence of miRNA transfection fades away because of the molecular degradation and some cellular adaptation or robustness mechanisms [15, 45, 46], then all methods cannot well predict miR-124, but still the NP-method ranks it best. Since the score *NPES* represents to what degree the miRNA-induced network perturbation can explain the gene differential expression levels, so we checked the *NPES* of miR-124 at every time point and found it got the maximum at 24 h (Figure 3B), and also we found most overlaps between consecutive LE target sets at 24 h (Figure 3C). Maybe at this time period, the impact of the miR-124 transfection gets sufficient and stable in the HepG2 cells, and thus all the methods are efficient in rediscovering the overexpressed miRNA from gene expression data.

**Table 4:** **Putative ranks of miR-124 at each time point after its transfection**

GSE6207.HCC.miR-124	4 h	8 h	16 h	24 h	32 h	72 h	120 h
NP-method	^a^2 (*)	1 (*)	1 (*)	1 (*)	1 (*)	1 (*)	15 (0.021)
GSEA	19 (0.081)	2 (*)	1 (*)	1 (*)	1 (*)	2 (*)	19 (*)
GR.GSEA	15 (0.04)	1 (*)	1 (*)	1 (*)	1 (*)	1 (*)	20 (*)

^a^Putative rank of miR-124 according to the normalized score (*p*-value of miR-124); *< 0.001.

At the same time, NP-method identified 188, 165, 172, 184, 168, 197 and 231 LE targets respectively at the seven time points (Figure 3C, see more details in Additional file 3). These LE targets mostly have very large fold change ratios among all the miR-124 targets and also they can generate the largest *NPES* score (Additional file 1: Figure S2), which means that these key targets are principally regulated by the miRNA and contribute a lot to the observed gene expression changes. There are 523 LE targets in total, including some known functional targets of miR-124. For example, the oncogenes *ROCK2* and *EZH2* that are direct targets of tumor-suppressive miR-124 in hepatocellular carcinoma [47], and the *IQGAP1* who is directly repressed by miR-124 in HCC cell lines and plays important functions in the cell adhesion and motility [48]. We analyzed the functional enrichment of all these 523 LE targets using the DAVID Functional Annotation Tool [49], and found they were significantly enriched in the protein localization, transport and signal transduction functions (Additional file 1: Table S2, adjusted *p*-value Benjamini ≤ 0.05). Besides, there were seven common genes shared by every time-point’s LE target set. These genes should be regulated by miR-124 all the time after its transfection. They are *CDK4*, *CD164*, *AMMECR1*, *RPIA*, *FAM177A1*, *RRBP1* and *MBOAT5*. The fold change patterns of these genes look very similar (Figure 3D), and according to the miRTarBase [36] the first five genes have been validated as direct targets of miR-124, so we guess *RRBP1* and *MBOAT5* are also its true targets in the HepG2 cells, which deserve further experimental verification.

### Uncovering the perturbed miRNA regulatory networks in colorectal cancer

**Figure 4:**
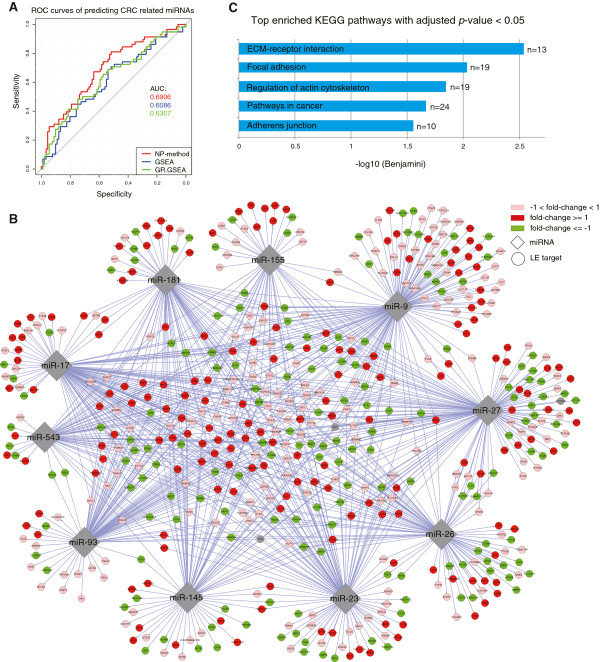
**Inference of perturbed oncogenic miRNA regulatory network in colorectal cancer. (A)** ROC curves of predicting CRC related miRNAs (families) using different methods. **(B)** The perturbed key miRNA regulatory sub-network in CRC. Genes are colored on the basis of their expression fold change. **(C)** Enrichment results of the 538 LE target genes in KEGG pathways. The number of LE genes included in each pathway is shown beside the bar.

Above analyses indicated that NP-method could identify the perturbed miRNAs as well as the leading-edge targets from the gene expression data of miRNA-perturbed cancer cell lines. Then we applied it on a gene expression dataset of clinical patient samples to infer the perturbed miRNA regulatory networks in colorectal cancer. The dataset GSE4107 profiled gene expressions from colonic mucosa cells of 12 patients and 10 healthy controls [50]. We firstly filtered low-expressed genes using the same strategy as the above time-course data analysis, and this left 15,996 genes. Then we calculated the gene expression fold change and respectively applied NP-method, GSEA and GR.GSEA to infer CRC associated miRNAs. From the results of each method, we obtained a list of miRNA families sorted in descending order according to the output normalized scores. In the meantime, we searched “colorectal cancer” in the miR2Disease, which is a manually curated database providing a comprehensive resource of miRNA deregulation in various human diseases, and got 89 CRC related miRNAs. In our work the miRNA family that contains at least one cancer miRNA was marked as a positive family, this produced 58 CRC associated miRNA families (See details in Additional file 4). Finally, we applied R package “pROC” [51] to calculate the sensitivity (i.e. true positive rate) and 1-specifity (i.e. false positive rate) along the miRNA family lists, and then drew the ROC curves and calculated their AUCs (Figure 4A). Results showed that NP-method had the largest AUC and thus best predicted the CRC related miRNA families.

From the results (More details can be found in Additional file 4) we selected 10 most significant miRNA families with *p*-value < 0.01 to be the perturbed key miRNAs, of which most had been reported playing important roles in the colorectal cancer progression. For example, the miR-27a [52], miR-17 [53], miR-155 [54], miR-9 [55] and miR-23a [52] can promote CRC cell proliferation, invasion or motility, and the miR-26b [56], miR-145 [57], miR-93 [58] and miR-23b [59] can inhibit CRC tumor growth, proliferation and induce apoptosis. Together with their LE targets we constructed a miRNA regulatory network in Figure 4B, where the 10 diamond nodes represent the miRNA families and 538 circular nodes are the LE target genes. The colors of genes characterize their expression fold change: red means significant up-expression (fold change ≥ 1), green means significant down-expression (fold change ≤ -1) and pink means not significant change. In the network, miR-9 has the largest out-degree and regulates 142 genes, which again highlights its importance in CRC development; while *ACVR1C*, also known as *ALK7*, has the largest in-degree of 7 and is down-expressed in the studied patient samples (log2 fold change −0.91), it is a type I receptor for the *TGFB* family of signaling molecules and has been found inducing apoptosis through activating *SMAD*s and *MAPKS* in tumor cells [60]. Then we also applied the DAVID tool to analyze the functional pathway enrichment of these 538 LE target genes, and found they were significantly enriched in 5 KEGG pathways (Benjamini ≤ 0.05, Figure 4C), which are all directly relevant to the cancer development and progression. All these results indicate that our method successfully finds out the key miRNA regulatory sub-network that is functionally perturbed or dys-regulated in colorectal cancer.

## Discussion

We hypothesize that the miRNA’s impact on target genes should propagate across the whole gene network and this impact could be better interpreted by integrating the differential expressions of all network genes not just the miRNA target genes. So we propose a novel network propagation based method (NP-method) to infer the perturbed miRNA regulatory networks using the differential expression information of global gene network. It executes random walk with restart (RWR) from the miRNA targets in the gene regulatory network to model the intracellular propagation effect of the miRNA perturbation, and meanwhile adopts a forward searching strategy to find the leading-edge targets that are principally regulated by the perturbed miRNAs and result in the observed global gene expression changes.

The analyses of the miRNA perturbed cell line data demonstrated that NP-method could detect perturbed miRNAs from gene differential expression profiles better than GSEA and GR.GSEA. Except for the prediction of pivotal miRNAs, another advantage is to extract the context-specific leading-edge targets for miRNAs at the same time. Even those low-key but functional targets, whose differential expressions are not much prominent but their down-stream gene expressions are significantly changed in response to the miRNA perturbation, can be discovered by our method. For example the miR-34a regulates *CUX1* in HCT116 cells. Besides, the analysis of time-course gene expressions from the miR-124 transfected cells revealed that the influence of miRNA perturbation in cells might be time-dependent and our method was more suitable for analyzing the perturbation effect at early time than other methods. In brief, NP-method can help to uncover the perturbed key miRNA regulatory networks in cellular processes of interest.

When analyzing the gene expression data of CRC patients, NP-method predicted the disease associated miRNAs better than other methods, which again proved its efficiency. And based on the results we successfully built a key miRNA regulatory sub-network that should be perturbed and play important functions in colorectal cancer. However, it is known that cancers are usually caused by multiple factors not just a single molecular deregulation like a miRNA overexpression or inhibition, so exploring the synergetic effect of a miRNA group should be more reasonable and meaningful. In this work, the NP-method considered the miRNAs or miRNA families as independent determiners of global gene expression changes and prioritized them according to the estimated network perturbation effect score (*NPES*). The top-ranked miRNAs are more likely to cause the observed gene differential expressions and are considered more important for the studied cellular process. In the future, we will take the miRNA cooperative regulation into account and try to infer the combination of miRNAs for better deciphering the miRNA-mediated cancer pathologies.

NP-method is not only applicable for analyzing miRNAs, but other problems about multiple interventions on a network are also theoretically appropriate. For example, some small-molecule drugs targeting several genes, proteins or enzymes in molecular networks [61]. So our approach can also be used to study the transcriptomic influence of the pharmacological interventions in cells. And with the increasing concerns on multi-target therapeutics [62, 63], we believe that our method can be further developed and help to design high-efficient combinatorial therapies for complex diseases.

## Conclusions

Here we developed a network propagation based method, which took advantage of the differential expression information of global gene network, to infer the perturbed functional miRNAs as well as their leading-edge targets. We demonstrated its reliability and usefulness on several cell line datasets and a clinical cancer dataset. Taken together, our method is a useful approach for studying the miRNA-mediated molecular mechanisms of complex biological processes.

## Electronic supplementary material

Additional file 1: Table S1: Putative ranks of experimentally perturbed miRNAs using different r in NP-method. **Table S2.** Top enriched GO terms for the 523 miR-124 LE targets (Benjamini < 0.05). **Figure S1.** Size of LE target subsets of the experimented miRNAs. **Figure S2.** Score and leading-edge targets of miR-124 at seven time points. Blue dot represents the absolute value of gene expression fold change, which is normalized by the maximum of all genes; Red dot stands for the *NPES* generated by NP-method, and the peak value is the optimal score and those targets appearing at and before this point are the leading-edge targets. (PDF 216 KB)

Additional file 2: **Results of analyzing the six miRNA perturbed cell line datasets.** (XLSX 128 KB)

Additional file 3: **Results of analyzing the time-course gene expression dataset and the original gene expression fold changes.** (XLSX 1 MB)

Additional file 4: **Results of analyzing the colorectal cancer dataset, miR2Disease supported cancer miRNAs and the original gene expression fold changes.** (XLSX 406 KB)
