# Supplementary material for: Inferring the perturbed microRNA regulatory networks from gene expression data using a network propagation based method
Source: BMC Bioinformatics. 2014 Jul 29;15(1):255. doi: 10.1186/1471-2105-15-255 (PMC4124158; doi:10.1186/1471-2105-15-255)
Supplement: Supplementary file 1 — Additional file 1: Table S1: Putative ranks of experimentally perturbed miRNAs using different r in NP-method. Table S2. Top enriched GO terms for the 523 miR-124 LE targets (Benjamini < 0.05). Figure S1. Size of LE target subsets of the experimented miRNAs. Figure S2. Score and leading-edge targets of miR-124 at seven time points. Blue dot represents the absolute value of gene expression fold change, which is normalized by the maximum of all genes; Red dot stands for the NPES generated by NP-method, and the peak value is the optimal score and those targets appearing at and before this point are the leading-edge targets. (PDF 216 KB) [file 12859_2014_6523_MOESM1_ESM.pdf]

Table S1 Putative ranks of experimentally perturbed miRNAs using different r in NP-method

Dataset, NP-method	r=0.1	r=0.2	r=0.3	r=0.4	r=0.5	r=0.6	r=0.7	r=0.8	r=0.9
GSE33420.CRC.mir-143	1	1	1	1	1	1	1	1	1
GSE18625.CRC.mir-145	1	1	1	1	1	1	1	1	1
GSE7754.CRC.mir-34a	51	49	47	46	45	43	42	42	41
GSE16568.OVCA.mir-22	1	1	1	1	1	1	1	1	1
GSE16569.OVCA.mir-30a	23	20	16	20	15	19	18	19	20
GSE16572.OVCA.mir-182	5	5	5	5	6	4	6	5	6

Table S2 Top enriched GO terms for the 523 miR-124 LE targets (Benjamini < 0.05)

Term	Count	PValue	Benjamini
GO:0033036~macromolecule localization	58	5.62E-06	0.013037
GO:0008104~protein localization	48	3.50E-05	0.026843
GO:0051179~localization	122	2.85E-05	0.032691
GO:0015031~protein transport	42	9.27E-05	0.035388
GO:0006810~transport	107	1.24E-04	0.035599
GO:0045184~establishment of protein localization	42	1.11E-04	0.03647
GO:0007264~small GTPase mediated signal transduction	23	7.99E-05	0.036587
GO:0048522~positive regulation of cellular process	82	6.56E-05	0.037549
GO:0007265~Ras protein signal transduction	12	2.24E-04	0.04634
GO:0051234~establishment of localization	107	1.87E-04	0.047207

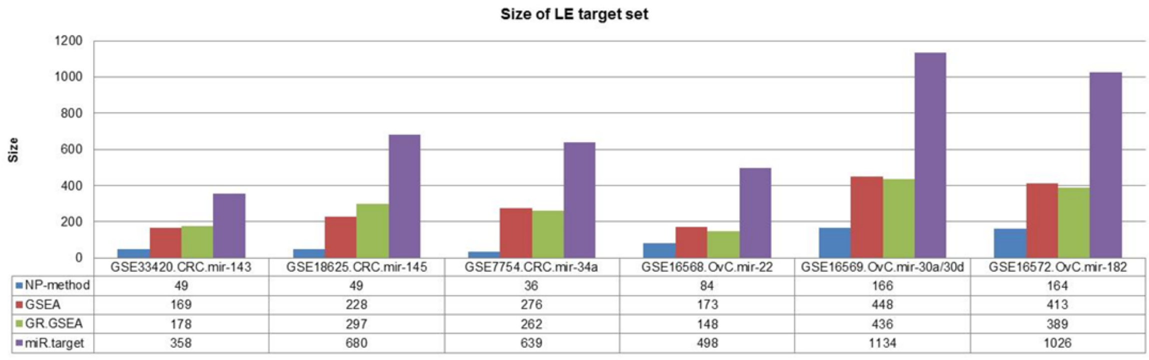

Figure S1 Size of LE target subsets of the experimented miRNAs

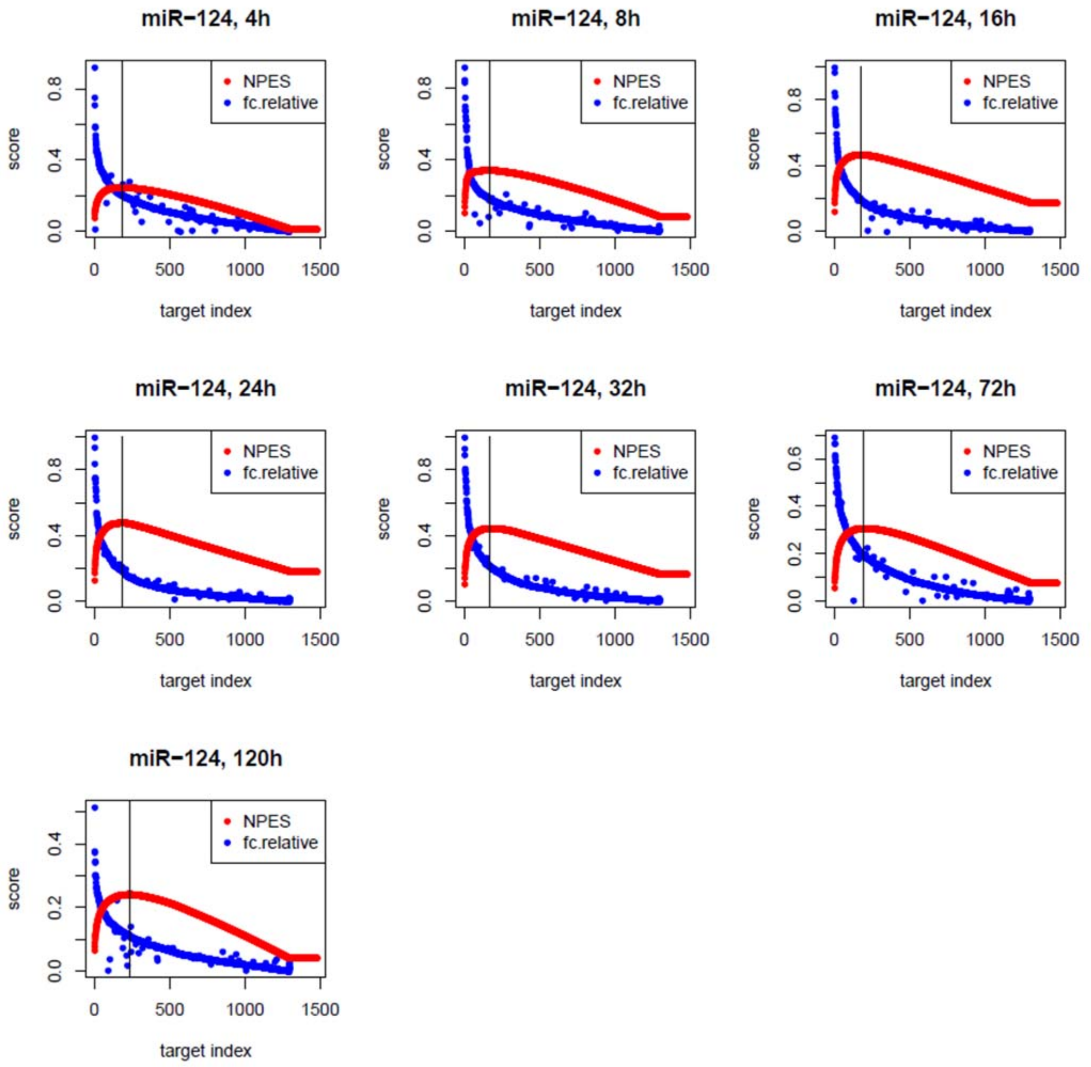

Figure S2 Score and leading-edge targets of miR-124 at seven time points. Blue dot represents the absolute value of gene expression fold change, which is normalized by the maximum of all genes; Red dot stands for the *NPES* generated by NP-method, and the peak value is the optimal score and those targets appearing at and before this point are the leading-edge targets.
